# Underutilization of Transcatheter Aortic Valve Replacement in Northern Plains American Indians with Severe Aortic Stenosis

**DOI:** 10.1007/s40615-023-01604-7

**Published:** 2023-05-02

**Authors:** Jon Roberts, Chase Gauthier, Luke Teigen, Hunter Row, Anne Sandstrom, Thomas Haldis, Cornelius Dyke

**Affiliations:** 1 Dakota School of Medicine and Health Sciences, University of North, Room 118, 1919 N Elm Street, Grand Forks, ND 58102-2416 USA; 2 Sanford Health, Fargo, ND USA

**Keywords:** American Indian, Structural heart disease, Transcatheter aortic valve replacement, Health disparities, Minority health

## Abstract

**Introduction:**

Transcatheter aortic valve replacement (TAVR) has overtaken surgical aortic valve replacement and revolutionized the treatment strategy for aortic valve replacement. Little is known on the disparities among minorities, especially American Indians (AI), undergoing this procedure. We explore TAVR outcomes to identify disparities at our institution.

**Methods:**

Retrospective chart review was completed on patients who underwent TAVR at a North Dakota community hospital between 2012 and 2021. There were 1133 non-AI and 20 AI patients identified (*n* = 1153). AI patients were identified by enrollment in nationally recognized tribes, Indian Health Service (IHS), or who self-identified as AI. Patient demographics, preoperative characteristics, procedural information, and outcomes were collected. United States 2020 census data was used for state-wide population racial percentages. Unpaired two tail *t* test assuming unequal variance and chi-squared tests were used to evaluate data and identify disparities between AI and non-AI.

**Results:**

AI presented at an earlier age (71 vs. 79; *p* = .001) with higher rates of diabetes (60% vs. 35%; *p* = .018) and history of smoking (100% vs. 60%; *p* ≤ .001) than Caucasian/white (C/W). The Society of Thoracic Surgery (STS) risk scores (3.2% vs. 4.6%; *p* = .054) and aortic valve mean gradients were lower among AI (42.8 mmHg vs. 47.5 mmHg; *p* = .010). For those deceased, AI had significantly shorter lifespans post-TAVR compared to C/W (374 days vs. 755 days; *p* = .004). AI from North Dakota had fewer TAVR procedures performed than expected (4 actual vs. 32 expected; *p* < .001).

**Conclusions:**

AI undergoing TAVR presented earlier, with higher rates of diabetes and smoking, lower STS risk scores, and lesser aortic valve gradients than C/W. The number of TAVR procedures performed on AI from North Dakota was lower than anticipated despite a nearly 10-year period and the disparities experienced by AI who could have otherwise benefited from TAVR.

## Introduction

Aortic stenosis affects up to 2.5 million people in the USA and is rapidly progressive after the onset of symptoms [1]. When severe, it usually leads to death in less than 2 years, making timely recognition and treatment critical [2]. Furthermore, aortic stenosis represents a major burden on our health care system costing an annual $10.2 billion [3]. There are two primary etiologies of aortic stenosis (AS): bicuspid congenital abnormality and calcific aortic valve stenosis. Calcific aortic stenosis has an estimated prevalence of 12.4% in the elderly population [1]. First performed in 1960, surgical aortic valve replacement (SAVR) was the sole and definitive treatment of severe aortic stenosis until the pioneering work of Cribier and colleagues who performed the first transcatheter aortic valve replacement (TAVR) in humans in 2002 [4]. Following successful clinical trials [5–7], TAVR has revolutionized the treatment of aortic stenosis, especially in the elderly population. Originally indicated in 2012 in the USA for patients at high risk for SAVR, the indications for TAVR have expanded and now include patients at all risk levels. TAVR also offers an effective treatment option for patients once considered too frail or with excessive risk for SAVR, further expanding the pool of patients receiving TAVR. TAVR has become the most common procedure performed for patients with aortic stenosis, outnumbering patients undergoing SAVR [8].

In accordance with regulation from the Centers for Medicare and Medicaid Services, TAVR procedures are only done at centers with experienced interventional cardiologists and cardiac surgeons comprising a heart team for the assessment and treatment of patients with aortic stenosis. This results in clustering of TAVR centers in urban or high-population areas and making access to care an issue for rural or disadvantaged populations [9]. Disparities in cardiovascular care, including cardiac surgical services, exist in American Indian/Alaskan Native (AI/AN) populations [10]. It is unclear whether AI/AN persons with severe aortic stenosis undergo TAVR at similar rates to non-American Indian/Alaskan Natives.

## Methods

All patients who underwent TAVR at a single center, community hospital in North Dakota between 8/10/2012 and 5/27/2021 were reviewed. This study was approved by the Sanford Health institutional review board. Patient demographics were identified on retrospective chart review and included age, self-identified race, distance from the procedural center, and pre-procedural comorbidities. The Society of Thoracic Surgeon risk score for individual patients was calculated based on demographics identified. The distance traveled to the hospital was estimated using a Google Maps search from the patients’ home zip code to the medical center. Procedural details and outcomes were recorded, including access point, mortality, and any procedural related deaths. The endpoint for follow up was June 6, 2021, and was used to assess mortality for all patients. For those deceased, lifespan after TAVR was recorded. Lifespan after TAVR was only compared between AI and C/W who were deceased.

Patient race was determined by self-identification or enrollment in recognized American Indian tribes. Patients were separated into five groups based on race: Caucasian/white (C/W; *n* = 1128), American Indian/Alaskan Native (AI/AN; *n* = 20), African American/Black (AA/B; *n* = 2), Asian (*n* = 1), and others who declined to racial identify (*n* = 2). The two largest racial groups identified, C/W and AI/AN, were the focus of this study. The use of the term “American Indians” (AI) throughout this manuscript describes this AI/AN group. Most patients in this study were residents of North Dakota (ND) or Minnesota (MN).

Categorical variables were analyzed using chi-squared tests. Continuous variables were analyzed using unpaired two tailed *t* tests assuming unequal variance. United States 2020 nationwide census data was used, specifically state-wide population racial percentages including “White alone” and “American Indian or Alaska Native alone.” These percentages were used to calculate the expected racial distribution of patients, based on their residency. For instance, the number of ND residents (*n* = 568) in this study was used in conjunction with US Census data on ND, to calculate the expected racial distribution of patients from ND. This analysis was done separately for ND, MN, and South Dakota (SD) residents. Patients from states other than ND, MN, or SD were grouped together (*n* = 16).

## Results

A total of 1153 patients underwent TAVR between 2012 and 2021, of which 1128 (97.8%) were C/W and 20 (1.7%) were AI. Patient demographics and clinical characteristics are detailed in **(**Table 1**)**. American Indians presented at a significantly earlier age (71 vs. 79; *p* = 0.001) and with higher rates of previous tobacco use (100% vs. 60%; *p* = 0.001) and diabetes (60% vs. 35%; *p* = 0.018). The severity of aortic stenosis, as assessed by pre-procedural echocardiographic mean transaortic valve gradient, was lower in AI than C/W (42.8 mmHg vs. 47.5 mmHg; *p* = 0.010). Preoperative STS risk scores were also lower in American Indians compared to C/W (3.2% vs. 4.6%; *p* = 0.054).

**Table 1:** Patient demographics and preoperative characteristics

Variable	C/W (*n* = 1128)	AI/AN (*n* = 20)	*p* value
Male:female	648:480 (57%:43%)	15: 5 (75%:25%)	.115
Height (m)	1.679 ± 0.101	1.693 ± 0.094	.531
Weight (kg)	86.07 ± 20.57	86.33 ± 20.06	.956
Age (years)	78.93 ± 8.74	71 ± 9.38	.001
Aortic valve mean gradient (mmHg)	47.5 ± 10.9	42.8 ± 6.9	.010
STS risk score	4.6 ± 3.6% (*n* = 1065)	3.2 ± 2.9% (*n* = 18)	.054
Distance traveled (miles)	115.4 ± 176.3	160.1 ± 163.3	.240
Hyperlipidemia	75.8% (*n* = 855)	90.0% (*n* = 18)	.140
Previous tobacco use (smoking or smokeless)	60% (*n* = 677)	100% (*n* = 20)	< .001
Current tobacco use	5.76% (*n* = 65)	15% (*n* = 3)	.083
Diabetes	34.57% (*n* = 390)	60% (*n* = 12)	.018
Diabetic treatment (insulin as primary or only treatment)	40% (*n* = 156)	58.33% (*n* = 7)	
Diabetic treatment (oral)	42.82% (*n* = 167)	33.33% (*n* = 4)	
Diabetic treatment (none/diet)	16.92%(*n* = 66)	8.33% (*n* = 1)	
COPD	17.64% (*n* = 199)	20% (*n* = 4)	.784
Heart failure	43.53% (*n* = 489)	55% (*n* = 11)	.298
CAD	67.55% (*n* = 762)	70% (*n* = 14)	.817
PVD	10.73% (*n* = 121)	20% (*n* = 4)	.187
CKD	27.93% (*n* = 315)	35% (*n* = 7)	.485
Stage 1	0.32% (*n* = 1)	0% (*n* = 0)	
Stage 2	5.71% (*n* = 18)	0% (*n* = 0)	
Stage 3	76.19% (*n* = 240)	42.86% (*n* = 3)	
Stage 4	7.62% (*n* = 24)	28.57% (*n* = 2)	
Stage 5	9.52% (*n* = 30)	28.57% (*n* = 2)	
Dialysis w/ CKD	8.89% (*n* = 28)	14.29% (*n* = 1)	.477
Previous CABG	19.86% (*n* = 224)	30% (*n* = 6)	.261
Previous stroke	14.63% (*n* = 165)	5% (*n* = 1)	.225
BMI	30.44 ± 6.44	30.19 ± 6.84	.870
Underweight (< 18.5)	0.71% (*n* = 8)	0% (*n* = 0)	
Healthy weight (18.5 – 24.9)	18.17% (*n* = 205)	25% (*n* = 5)	
Overweight (25 – 29.9)	30% (*n* = 393)	30% (*n* = 6)	
Obese (30 +)	46.28% (*n* = 522)	45% (*n* = 9)	
AVG. FVC % predicted	82.61 ± 20.78% (*n* = 937)	81.44 ± 24.43% (*n* = 16)	.851
AVG. FEV1% predicted	83.11 ± 39.11% (*n* = 937)	84.75 ± 29.83% (*n* = 16)	.831
AVG. FEV1/FVC	71.36 ± 11.25 (*n* = 937)	75.25 ± 11.26 (*n* = 16)	.190

*COPD* chronic obstructive pulmonary disease, *CAD* coronary artery disease, *PVD* peripheral vascular disease, *CKD* chronic kidney disease, *CABG* coronary artery bypass graft, *BMI* body mass index, *FVC* forced vital capacity, *FEV1* forced expiratory volume 1 s

**Fig. 1:**
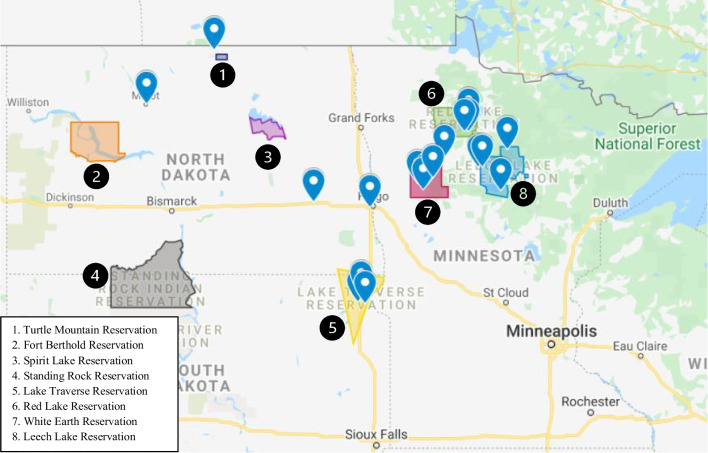
Geographic distribution of AI patients. Geographic distribution of American Indian patients and federally recognized reservation. All patients are represented; each pin may represent more than one patient

Geographic differences existed between American Indian and non-American Indian patients undergoing TAVR. The geographic distribution of AI patients is presented in (Fig. 1). Patients were primarily from North Dakota (49%) and Minnesota (47%). Although not statistically significant, American Indians traveled further for TAVR than C/W (160 miles vs 115 miles; *p* = 0.240). For patients from North Dakota, 98.8% were C/W versus 0.7% AI, and of those from Minnesota, 97.8% where C/W versus 2.2% AI. Racial demographics in ND, MN, and SD, based upon the 2020 census, are detailed in (Table 2). When comparing the expected TAVR procedures volumes based upon population estimates, American Indians from North Dakota had fewer TAVR procedures performed than expected (4 actual vs. 32 expected).

**Table 2:** TAVR cases by state with census data and expected AI/AN cases

Variable	TAVR cases					Census data			
	ND residents	MN residents	SD residents	Other state residents	Total	ND census data	MN census data	SD census data	US census data
C/W	568 (98.78%)	532 (97.79%)	13 (81.25%)	15 (93.75%)	1128	86.9%	83.3%	84.6%	76.3%
AI/AN	4 (0.70%)	12 (2.21%)	3 (18.75%)	1 (6.25%)	20	5.6%	1.4%	9.0%	1.3%
AI/AN expected # of cases	32	8	1	0	41 (*p* < .001)				

Abbreviations: *ND* North Dakota, *MN* Minnesota, *SD* South Dakota

Short-term outcomes were similar between groups, and both groups had similar lengths of hospital stay (3.7 days in C/W vs. 3.2 days in AI; *p* = 0.975) (Table 3**)**. One-year mortality was numerically higher in AI compared with C/W, but did not reach statistical significance (20.0% vs. 11.9%; *p* = 0.268). The average follow-up time after TAVR was of 883 days. For those deceased, lifespan after TAVR was recorded. Post-procedural average lifespan for those deceased was significantly reduced in American Indian patients (755 days for C/W vs. 374 days for AI; *p* = 0.004).

**Table 3:** Procedural details and outcomes

Variable	All races	C/W	AI/AN	*p* value
Transfemoral access	1000	978	18	
Transaxial access	53	53	0	
Other access	8	26	1	
Length of hospital stay	3.18 ± 4.63	3.17 ± 4.65	3.20 ± 3.76	.975
Mortality rate (as of 6/20/21, median follow-up time of 883 days)	32%	32%	35%	.743
1-yr mortality	12% (*n* = 141)	12% (*n* = 134)	20% (*n* = 4)	.268
Days of life after procedure (for those deceased)	741 ± 669	755 ± 673	374 ± 235	.004

## Discussion

Fewer AI patients underwent TAVR than expected based on the AI population within our region, partly due to underrepresentation. Therefore, this study lacks sufficient power to identify outcome differences between AI and C/W patients. As the largest provider in the Northern Plains, the paucity of AI patients was unexpected and suggests either a disparity in diagnosis or referral for treatment. For AI patients who did undergo TAVR, significant differences did exist, including higher rates of active tobacco use and diabetes mellitus. Additionally, AI patients presented with lower STS scores, attributed to a younger age at presentation and lower pre-TAVR mean gradients. AI patients also survived fewer days after TAVR than non-AI patients, although this trend was not significant.

The geographic distribution of patients undergoing TAVR is represented in Fig. 1. The Upper Midwest is a rural region, and patients in both groups traveled significant distances (more than 115 miles on average) for TAVR. American Indians, however, traveled further for treatment of their severe aortic stenosis than C/W (160 vs. 116 miles; *p* = 0.240). These geographic differences are likely due to clustering of American Indian patients on or near recognized reservation land and are health care disparities generally, as local access to specialty care is constrained. Structural conditions of patient lives, including where they were born, proximity to care, access to transportation, and access to providers, have been demonstrated to have a significant impact on health and contribute to healthcare disparities seen in rural and disadvantaged populations especially [11]. American Indians also presented at an earlier age for TAVR and were significantly more likely to use tobacco and have diabetes mellitus treated with insulin **(**Table 1**)** than non-American Indians. Preoperative STS risk scores were lower in American Indians (3.2% vs. 4.6%; *p* = 0.054), likely because of younger presentation.

In an earlier study of American Indians undergoing coronary artery bypass surgery in our institution, rates of surgery were consistent with population estimates; one would expect rates for TAVR in American Indians would be similarly consistent with population incidence [10]. The reason for this underutilization is unclear but may lie in the generally elective evaluation of patients with structural heart disease as opposed to acute coronary syndromes, in which patients are seen and transferred more urgently. Patients with ischemic heart disease undergoing coronary artery bypass usually present acutely to emergency facilities with acute coronary syndromes and are rapidly triaged to larger centers such as ours. On the other hand, aortic stenosis presents insidiously, and patients are usually worked-up electively, potentially leading to a greater chance for patients to be lost to follow-up or simply never evaluated due to access limitations, financial constraints, under-insured status, or other reasons. Additionally, whether American Indians are underdiagnosed or not is unclear, but highly possible as specialist care and sophisticated testing with echocardiography and cardiac catheterization, among others, is necessary to diagnose and follow patients with aortic stenosis. The under-representation of American Indians receiving TAVR is an example of how structural healthcare problems and societal problems are manifested in individual patients, resulting in under treatment and preventable death.

When severe aortic stenosis in American Indians is diagnosed and American Indian patients undergo TAVR, short-term outcomes are good. We found that short-term and procedural outcomes after TAVR were similar between groups, suggesting that TAVR is safe and effective for American Indians and non-American Indians with severe aortic stenosis. While in-hospital outcomes are similar, however, outcomes at 1 year may be worse in American Indians after TAVR. While recognizing that our study underpowered to detect mortality differences, 1-year mortality in American Indians was nearly double the 1-year mortality in C/W (20.0% vs 11.9%; *p* = 0.268). Additionally, for patients who died, the interval between procedure and death was nearly 50% shorter in American Indian patients. These signals of poor outcomes after TAVR are concerning and need confirmation; unfortunately, larger studies of TAVR in AI/AN patients are lacking.

Potentially, increased smoking rates and diabetes may contribute to increased mortality after TAVR. We hypothesize, however, that additional societal and structural factors such as geographic distance and reduced access to follow-up care are more likely explanations for reduced early survival. TAVR patients need medical care after the procedure for heart rate and rhythm control, anticoagulation, and prosthetic valve assessment. Limits to this care will affect outcomes. It has been previously reported that American Indians are less likely to see a medical doctor or have a usual source of care. In surgical patients, access to care has been demonstrated to affect outcomes [12]. Identifying “surgical deserts” such as rural areas, reservations, or Indian Health Service facilities prospectively may identify patients at risk for inadequate follow-up after procedures, including TAVR [13]. Other factors influencing patient health such as employment and housing status, health care literacy, and insurance coverage are also important [14].

In a similar population of American Indians undergoing coronary artery bypass surgery, we previously demonstrated that American Indians were less likely to receive guideline-directed medical care after cardiac surgery [15]. We hypothesize something similar is occurring in patients with aortic stenosis before correction and also occurs after TAVR. The decreased survival in American Indians 1 year after TAVR highlights the importance of the linkage between tertiary care facilities where these highly technical procedures are done and patient’s home community and providers. Increased attention to post-procedural care to mitigate these survival differences is an area of important further research.

## Conclusions

American Indians underwent TAVR less frequently than anticipated based on the population data of our region. Northern Plains American Indians who did undergo TAVR for severe aortic stenosis presented at a younger age, with a higher prevalence of diabetes and previous tobacco use, and lower STS scores and pre-TAVR mean gradients compared to non-American Indians. Reasons for the under-representation of American Indians in our population of patients undergoing TAVR are unclear, although inadequate evaluation, bias, and lack of funding for preoperative testing are possible. Unfortunately, American Indians are under-represented in prior clinical trials and current databases of patients undergoing TAVR, including ours, making outcome comparisons difficult.

## Data Availability

The data used in this study are not publicly available due to institutional policies that prevent open sharing. However, the data may be made available upon request, subject to appropriate data use agreements and ethical clearance.
